# Application of Chicory Root Inulin and Commercial Inulin in Various Food Products and Impact on Their Quality

**DOI:** 10.3390/foods15172979

**Published:** 2026-08-25

**Authors:** Iva Ostrun, Vesna Mihatov, Anita Pichler, Antun Jozinović, Ines Banjari, Mirela Kopjar

**Affiliations:** 1 Faculty of Food Technology Osijek, Josip Juraj Strossmayer University of Osijek, Franje Kuhača 18, 31000 Osijek, Croatia; iostrun@ptfos.hr (I.O.); anita.pichler@ptfos.hr (A.P.); ajozinovic@ptfos.hr (A.J.); ibanjari@ptfos.hr (I.B.); 2 Franck dd, Vodovodna Ulica 20, 10000 Zagreb, Croatia; vesna.mihatov@franck.hr

**Keywords:** chicory root inulin, commercial inulin, degree of polymerization, food applications

## Abstract

Chicory root has been used in traditional medicine, as a vegetable, and as a coffee substitute, and over the years it has become popular for its inulin content, becoming one of the major commercial sources of inulin for food applications. This review is intended to update knowledge in the field of chicory root inulin application and commercial inulin, as its use has been on the rise over the past decade. The techno-functional behavior of inulin in foods is primarily related to its degree of polymerization, and it can be used in various products such as dairy, meat, bakery products, and confectionery. In addition to providing basic information about the structure and properties of inulin, this review explores chicory root and commercial inulin’s application in the aforementioned categories of food products by suggesting the appropriate amount of added inulin to achieve the desired properties, the form in which it is used, its functional role, key processing conditions, and its limitations. Inulin can be used to improve the nutritional value of foods, modify texture, replace fat and sugar, and enhance probiotic viability; however, its impact on sensory characteristics cannot be neglected.

## 1. Introduction

Chicory root (*Cichorium intybus* var. *sativum*) is a plant species commercially cultivated for industrial purposes and is botanically classified in the Asteraceae family. Throughout history, chicory root has been used in traditional medicine and is also commercially utilized as a vegetable, while roasted root is often used as a coffee substitute. Chicory remains popular as a coffee substitute and has gained attention for its inulin content. Consequently, it has become one of the major commercial sources of inulin for food applications [1]. Chicory yields up to 45 tons per hectare [2] and contains approximately 17% inulin in fresh mass; thus, it is considered an important industrial source of inulin which is known for its health benefits as well as technological properties [3]. Chicory chemical composition differs depending on plant organs. The leaves contain high concentrations of flavonoids and other phenolic compounds, and are particularly known for their richness in chicoric acid, while the chicory root contains large amounts of storage carbohydrates almost exclusively as inulin, and secondary metabolites [1,4,5,6]. In addition to inulin as the primary compound, the root contains 9–15% reducing sugars and lower amounts of sucrose, proteins, cellulose, pectins, B complex vitamins and vitamin C and minerals [1,6,7] as well as a diverse range of bioactive compounds, including chlorogenic and protocatechuic acids, and sesquiterpene lactones [4,7]. These constituents as well as inulin play a role in the technological and sensory properties of foods in which they are added [4,8,9].

Inulin is a dietary fiber that is resistant to enzymatic digestion in the upper gastrointestinal tract [10]. The majority of inulin reaches the colon in intact form, where it undergoes fermentation by the existing gut microbiota, producing short-chain fatty acids (SCFAs) that are associated with beneficial effects on intestinal and metabolic health [3,10,11]. Some of these benefits involve improvement of glycemic response and mineral absorption, as well as alterations of lipid metabolism [12,13]. The techno-functional behavior of inulin in foods is primarily related to its degree of polymerization (DP) defining its potential application in foods [11,13,14,15,16]. Long-chain inulin is characteristic for forming gel-like structures and can mimic fat in foods [12,13,14,15], while shorter-chain fractions are more soluble, provide mild sweetness, and can be used for sugar reduction [11,12,13,14,15].

Currently, chicory root continues to be used as a caffeine-free coffee substitute and as a significant source of inulin, a food additive that improves technological properties and supports the formulation of high-value foods. Interest in the application of inulin in different food products has been steadily increasing, and over the years many studies have been conducted to improve food product quality and provide new insights in this field. Consequently, there was a need to summarize newly available data on this topic. Existing reviews on the application of inulin in foods either include inulin from different sources, are out of date, focus on extraction procedures, techno-functional properties, or concentrate mainly on health benefits, such as the prebiotic effect of inulin provided through food products [17,18,19]. For comparison, the most recent review related to inulin [19] summarized several inulin-related topics. It dealt with inulin’s structure, different sources of inulin, extraction techniques, its physicochemical properties (mostly related to gelling), and a concise summary of health benefits and nutritional aspects. That manuscript also included technological aspects, more precisely the application of inulin in food systems for sugar replacement, fat replacement, fiber enrichment, and other uses. The authors emphasized that chicory root generally has high yields and good cold tolerance, making it the most suitable and widely used crop for large-scale inulin extraction and consequently implying its broad use. Therefore, the existing reviews were not strictly focused on the application of inulin in food products, with a focus on all quality properties, clear presentation, and easier comparison of the use of inulin in powder and gel form. In this review, we concentrated on a detailed description of the application of inulin to overall quality in various products and, wherever scientific data allowed, we compared the impact of inulin from other sources with chicory root inulin. Thus, we focus our literature review on inulin obtained from chicory root, commercial inulin (since chicory root is the main source of inulin), and chicory extracts containing inulin. Because many new findings have been reported in the last decade, the field of inulin applications has expanded across different food products, and more specific information has been obtained, making it necessary to collect new data and update previous reviews. More detailed information is provided regarding the amounts of inulin and the form in which inulin can be used, as well as its positive or negative effects on overall product quality depending on the product type.

This extensive review examines the functional and technological properties of chicory root inulin, commercial inulin, and chicory extracts containing inulin and their application in food products, additionally emphasizing the chemistry behind it. The findings are presented in detailed tables to provide clearer insight and to facilitate comparison of how different amounts of inulin in different forms affect various quality aspects of food products, thereby highlighting their industrial relevance.

## 2. Methodology of Literature Screening

For this review, relevant scientific publications were identified through Google Scholar searches and by screening the reference lists of relevant review and primary articles. The initial search was conducted in late February 2026 and was subsequently updated iteratively during manuscript preparation as specific food applications and research topics were identified. No formal publication-date restriction was applied, although recent literature was prioritized and older publications were retained when relevant. Searches included general terms such as “chicory root inulin,” “inulin in food industry,” and “inulin food application,” as well as product-specific combinations such as “inulin in yogurt,” “inulin in cheese,” “inulin in ice cream,” “inulin in sausages,” “inulin in bakery products,” and “inulin in chocolate.” Additional terms related to fat and sugar replacement, texture modification, degree of polymerization, process stability, and cryoprotection were used individually or in combination with the Boolean operators AND and OR. Search terms were adapted and expanded iteratively according to the food application being investigated. Publications were considered relevant when they addressed chicory-root-derived inulin, commercial inulin or included in comparisons with other sources, with experimental studies prioritized for the analysis of food applications. Screening was based on title, abstract, and, where appropriate, the conclusions and full text. A total of 105 publications were used in the review. Data from food application studies were extracted according to product, inulin type/form, inulin amount, technological/functional role, key processing conditions, positive effects, limitations, and best-performing formulation, with DP recorded where reported. No formal risk-of-bias or evidence-quality assessment was performed; findings were interpreted primarily based on the reported experimental results, methodological information, and consistency of findings across studies.

## 3. Inulin Structure and Properties

Inulin, a water-soluble carbohydrate from the fructan family, is most commonly obtained commercially from chicory root [14]. Inulin content is typically reported as around 40–60% of dry weight, while higher values (up to approximately 70%) have been observed found in selected industrial cultivars [4,6,14].

### 3.1. Structural Characteristic

Structurally, inulin is a linear polysaccharide composed of fructose units linked by β-(2→1) glycosidic bonds, usually ending with a glucose moiety at the reducing end [16,20]. Unlike most plant polysaccharides that are composed of pyranose rings, inulin is characterized by the fact that it consists of fructofuranose units. These molecules adopt a more flexible conformation, which consequently affects their physicochemical properties [21,22]. Natural inulin is, in most cases, not a homogenous molecule but a polydisperse mixture of fructan chains of different polymerization length that vary depending on the plant source and harvest time [23]. According to the length of the chain, inulin can be classified as either an oligosaccharide or a polysaccharide [22].

**Figure 1:**
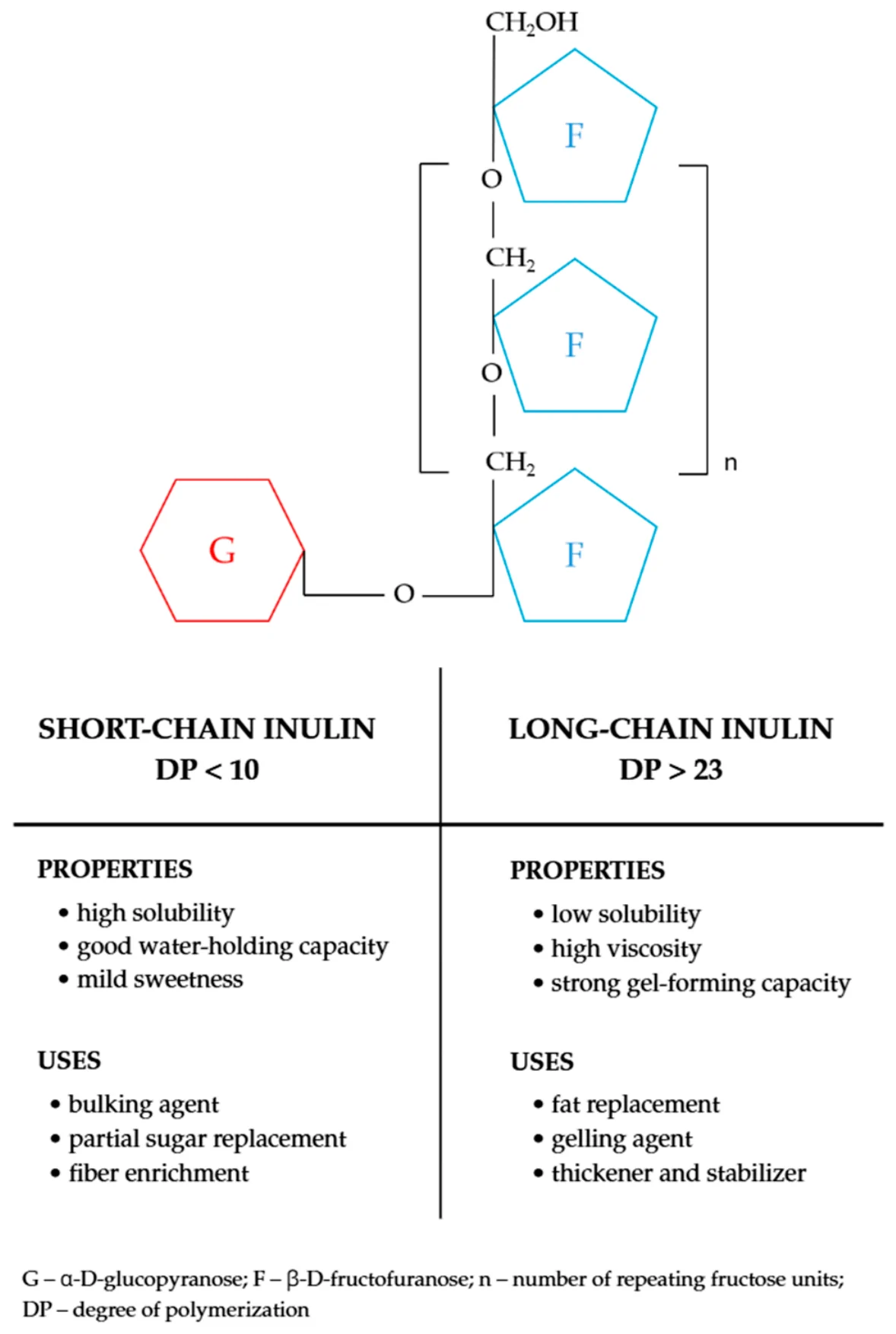
A schematic illustration of inulin structure, its degree of polymerization (DP) and the main properties and applications of short-chain and long-chain inulin.

As a native compound, inulin has DP from to 2 to 60; since its functionality depends on the degree of polymerization (DP), it is categorized into short-chain (DP < 10), and long-chain (DP > 23) fractions [5,12,20]. The distribution of the length of the chains significantly determines its physicochemical properties, especially its solubility in water, which increases with the decrease in the degree of polymerization [22,23]. Short-chain inulin, or oligofructose, is highly soluble and provides approximately 30–50% of the sweetness of sucrose, making it suitable for sugar-reduction [9,11]. Although short-chain fractions contribute to sweetness, their sweetening power is insufficient to replace sucrose on their own [15]. In contrast, long-chain inulin has lower solubility and higher viscosity [12,13]. At concentrations of 20–40%, it forms a three-dimensional network of insoluble microcrystalline particles, creating a gel that retains water and mimics the mouthfeel of fat, serving as an effective fat mimetic in dairy products such as yogurt and low-fat cheese [12,13,15,24]. It can be used in these products to reduce the energy value of food due to its low caloric value [24]. Figure 1 summarizes the relationship between the molecular structure of inulin, its degree of polymerization, and the characteristic properties and applications of short- and long-chain fractions.

### 3.2. Fermentation

From a nutritional perspective, β-(2→1) glycosidic bonds make inulin resistant to hydrolysis by human digestive enzymes, since these enzymes are primarily specific for α-glycosidic bonds [25]. Consequently, inulin passes through the small intestine undigested and reaches the large intestine, where it undergoes hydrolysis by the intestinal microbiota. Simple sugars are formed that are fermented into short-chain fatty acids (SCFAs) and microbial biomass [26]. In this process, inulin serves as a selective substrate for the growth of beneficial bacteria, especially the genera *Bifidobacterium* and *Lactobacillus* [5,11,16,27,28].

Anaerobic fermentation primarily produces acetate, propionate and butyrate [20], with SCFAs representing the main source of energy for colonocytes and providing approximately 60–70% of their energy requirements [26]. In addition, they contribute to maintaining the integrity of the intestinal barrier and regulating the immune response, lipid metabolism and glucose homeostasis [29]. Fermentation lowers the pH in the large intestine, creating unfavorable conditions for the growth and reproduction of pathogenic microorganisms, while increasing the bioavailability and absorption of minerals, especially calcium and magnesium [11,19]. The physiological effect is also dose-dependent, so studies have shown that a daily intake of only 5 g has prebiotic effects, while intake of approximately 15 g per day is associated with increased calcium absorption [15,30,31]. In addition to its individual effects, inulin may interact with other indigestible carbohydrates, improving microbial fermentation and affecting the composition of the gut microbiota, with consequent change in SCFA production [5].

### 3.3. Blood Glucose Regulation

Because of its resistance to enzymatic degradation and its very low glycemic impact, inulin is often used in foods with a low glycemic index, especially those intended for people with (pre)diabetes [19]. There are indications that chicory-derived inulin may improve insulin sensitivity, particularly in people with impaired fasting glucose, suggesting potential metabolic benefits for people at risk of type 2 diabetes [8,32].

### 3.4. Cryoprotection

Acting as a natural cryoprotectant of plant origin, inulin significantly contributes to the stability of frozen foods. By forming hydrogen bonds with water molecules, it limits the formation of large ice crystals and reduces fluid loss during defrosting. This effectively maintains the textural integrity of protein-rich matrices, including meat products [33]. Furthermore, the effectiveness of these antifreeze effects depends on chain length. Long-chain inulin in various products, such as silver carp surimi, frozen desserts, and processed meats, shows exceptional efficacy, often outperforming traditional cryoprotectants, such as sucrose, in minimizing thawing loss, preserving gel stability and water-holding capacity [19].

### 3.5. Gel-Forming Property

The gel-forming properties of inulin are highly important for adapting the textural characteristics of food products to which inulin is added, as well as for its ability to serve as a fat replacer. When long-chain inulin is added to water, depending on the inulin content and the water temperature, part of the inulin dissolves. The polydisperse oligosaccharides that dissolve form rods of various lengths, and upon cooling, recrystallization of the dissolved molecules occurs. First, inulin molecules with larger DP undergo recrystallization because they are less soluble in water, while molecules with lower DP accumulate in the outer regions of the developed crystallites. Further crystallization leads to the formation of aggregates of inulin crystallites, which act as primary particles in the gel network. These primary particles attract each other through van der Waals forces, entrapping water in the interspaces and resulting in the formation of a particle gel network. For different types of inulin, depending on their average DP, the gel network varies in particle size and packing density. These variations have a significant effect on the physical properties of the final inulin gels [19,34,35,36]. The crystallites in the inulin gel network are approximately 100 nm in diameter and, during aggregation, form larger aggregates of 1–5 μm, which trap a large amount of water in the network. The characteristics of this gel network resemble those of a network of fat crystals in oil, thereby reproducing the rheological and sensory functions of lipids [19,34]. Because this organization of the gel network closely resembles the crystalline structure of fats, it also has the ability to provide spreadability, creaminess, and mouthfeel [19,34,37]. Gel formation was schematically presented in manuscripts by Joshi et al. [34] and Canazza et al. [19].

### 3.6. Processing Stability

The stability of inulin during food processing depends on several factors, including the degree of polymerization, pH, temperature, heating time and the composition of the food matrix. In neutral and slightly acidic systems, inulin is chemically stable and retains its functional properties even during heat treatment. However, at pH ≤ 4, its stability decreases significantly, especially at temperatures above 60 °C and with longer heating times, when hydrolysis of β-(2→1)-glycosidic bonds occurs and the degree of polymerization decreases. Consequently, the gelling ability of inulin is impaired, and its technological properties change [12,19,38].

Therefore, the use of inulin is limited in highly acidic products that undergo intensive heat treatment, while in products with a pH ≥ 5 it remains stable even when heated to 100 °C [38]. The degree of polymerization also affects stability during processing. In the bakery industry, long-chain inulin may support gluten-network structure and increase bread volume, while short-chain fractions are more sensitive to the Maillard reaction and non-enzymatic browning due to the greater number of reducing ends, improving crust color and flavor produced by heating. This characteristic represents a technological advantage in bakery products and roasted chicory coffee substitutes, but at the same time, short-chain fractions are more susceptible to thermal degradation under acidic conditions [8,12].

Choosing the right degree of polymerization is also important in other food products. For example, in pasta, inulin with a high DP is generally preferable because it more effectively maintains the structural integrity of the protein and starch matrix, thus contributing to product strength and reducing losses during cooking compared to fractions with a lower DP [39].

In addition to chemical stability, the rheological properties of inulin are also important. The stability of gels primarily depends on temperature, concentration, and interactions with other hydrocolloids, such as gelatin, alginate, or carrageenan [11,24]. These interactions can further improve the rheological properties of emulsified and aerated products [14,40].

## 4. Food Applications

**Figure 2:**
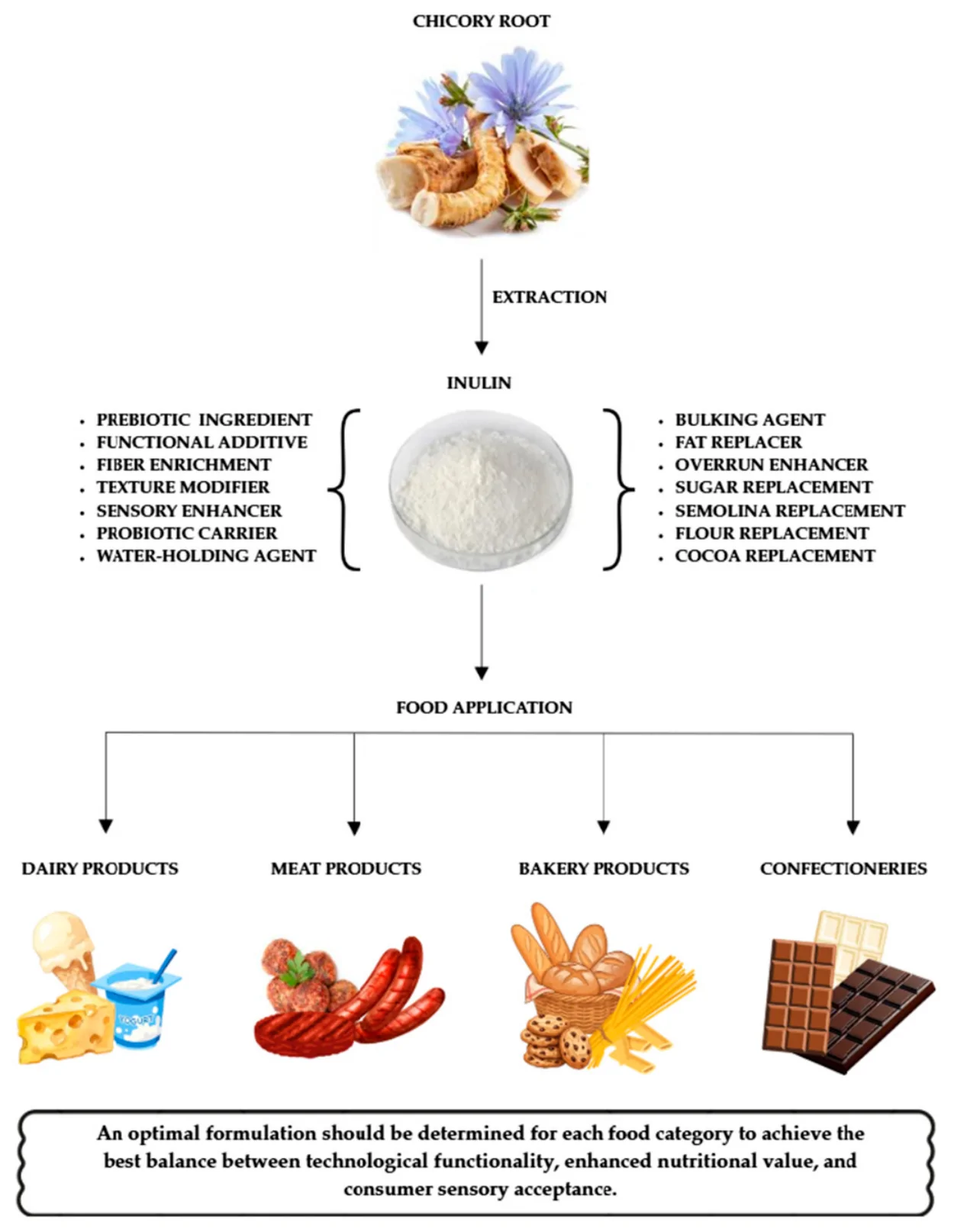
A schematic illustration of inulin’s application in food products and its impact on quality.

The previously mentioned properties of inulin enable its application in various food products to improve their quality and to develop new products. Despite the proven technological and nutritional benefits of chicory-derived ingredients, successful product development still requires careful optimization of formulation, since maintaining technological functionality alone does not guarantee consumer acceptance. Studies consistently show that moderate inulin incorporation provides the best balance between technological performance and sensory quality [41,42]. Summarizing the current knowledge, the available scientific evidence indicates that inulin from chicory can simultaneously improve the technological properties and nutritional value of products [14,24,43,44]. In the following text, the use of inulin in the dairy, meat, bakery and confectionery industries will be discussed, with an overview of its impact on these products and an estimation of the appropriate amount required to formulate high-quality products. Inulin’s application in food products and its impact on quality are schematically illustrated in Figure 2.

### 4.1. Dairy Products

Chicory inulin finds one of its most important applications in the dairy industry, due to its combined technological and nutritional value in liquid, semi-solid and fermented products [14,28,40,45]. A review of the multiple functionalities of inulin from chicory in dairy products is summarized in Table 1, Table 2 and Table 3. In general, inulin contributes to fat reduction through its replacement, increased dietary fiber content, stabilization of probiotic cultures and preservation of desirable rheological and sensory properties of dairy products. Because of its versatility, it supports the development of next-generation functional dairy products tailored to the growing consumer demand for healthier formulations [14,28,40,45]. However, adequate selection of the amount of inulin is essential and primarily depends on the product type, as excessive amounts can impair the rheological, structural and sensory properties of the final product.

In high-moisture and whey-based systems, it has been shown that inulin improved gel structure stability, modified protein–polysaccharide interactions and contributed to better rheological properties. Additionally, it improved water-holding capacity and preserved the product’s structural integrity [19,40,45,46]. These attributes enable its effective application in various products not only in frozen desserts, but also in yogurts, milk spreads, cream cheeses, and mousses [14,40]. In fermented dairy products, inulin contributes to texture stability by enhancing water-holding capacity and reducing syneresis due to the formation of a denser and more homogeneous gel structure, which positively affects the rheological properties of the product [46]. Long-chain inulin fractions, under the action of shear form a microscopic crystal network in aqueous media and therefore act as effective fat replacers. This structure enables a creamy texture and mouthfeel similar to that of a full-fat product, while allowing the development of reduced-fat products with acceptable sensory and structural properties [14,19,40].

#### 4.1.1. Application in Yogurt

The addition of inulin affected acidification kinetics during fermentation with intensity depending on complete matrix. Yogurts enriched with chicory ingredients have shown that low-to-moderate additions do not cause significant changes in pH or titratable acidity, indicating that the activity of starter cultures remains largely preserved within the usual formulation ranges [47]. It is important to keep in mind that the chemical stability of inulin decreases at acidic pH values below 4 as heating temperature and time increase. In contrast, under neutral and alkaline conditions, inulin is chemically stable regardless of the previously mentioned parameters. Consequently, inulin has limited applications in acidic foods, especially when thermal treatment is above 60 °C, whereas increasing the pH above 5 ensures its stability up to 100 °C [38].

The effectiveness of inulin in frozen and fermented dairy products, particularly yogurt, depends on finding the optimal amount that ensures rheological stability without compromising sensory properties. In yogurt production, low-to-moderate amounts (1–2%) were usually used, which improved viscosity, creaminess and water-holding capacity, effectively reducing syneresis [10,46,48]. For example, Sarwar et al. [46] found that a 1% inulin content together with 0.5% *Saccharomyces boulardii* yeast in synbiotic yogurt ensured maximum sensory acceptability and synthesis of aroma volatile substances, while increasing the amount to 2% resulted in a decrease in acceptability. Such limitations were also indicated by El-Kholy et al. [48] in low-fat yogurts. They concluded that the addition of up to 1% inulin improved texture and prevented whey separation, while a dose of 2% degraded the microstructure of the gel, increasing the porosity of the mesh and reducing its strength. In fermented dairy products, the structural effects of inulin can be further enhanced by combining it with other hydrocolloids. For example, combining chicory or artichoke inulin with maltodextrin in a 1:2 ratio (at a total of 2%) produced a denser gel structure, accelerated fermentation, and minimized whey separation during storage [28]. Although pure inulin effectively reduced syneresis, its combination with maltodextrin results in a significantly greater increase in apparent viscosity and overall stability of the system. Scientific data indicate the existence of a dose-dependent sensory threshold for inulin supplementation. Although increasing the amount of inulin (3–15%) progressively improved viscosity and firmness and reduced syneresis in natural yogurts, it also increased stickiness, demonstrating that technological improvement did not necessarily translate into enhanced sensory quality [10]. Consequently, such high-enrichment approaches were generally unsuitable for conventional commercial formulations, as excessive fortification in highly hydrated yogurt systems produced an overly dense and sticky texture, ultimately reducing consumer acceptability [46,48].

#### 4.1.2. Application in Cheese

The incorporation of inulin into the protein network of cheese takes place without direct interaction with casein micelles, which has a positive effect on the rheological properties of the product resulting in softer texture while whey separation is reduced to a minimum [40]. In cheesemaking, inulin is physically incorporated into the protein gel network, where it acts as a textural modifier and enhances the water-holding capacity of the cheese. Its effectiveness largely depends on interactions with milk proteins (primarily casein) and the spatial organization of the cheese matrix. In low-fat synbiotic soft cheese produced by ultrafiltration (UF) technology, a relatively high inulin amount (5–7%) effectively replaced milk fat, improving moisture retention, creaminess, probiotic viability and sensory quality. However, these benefits were accompanied by a slight reduction in pH and protein content [49]. A similar observation has been made in imitation of cheeses such as mozzarella, where replacing 10% to 50% of vegetable fat with inulin (optimal inulin content of 7.2% in the formulation) increased water holding capacity and stretchability, while extremely high amounts of inulin lead to excessive hardness and loss of cohesiveness compared with a full-fat control sample [50].

In cottage cheese, a low amount of inulin (1%) successfully replaced milk fat (4%) without compromising sensory quality, while a higher amount (2–3%) negatively affected sensory and physical properties [51]. These studies collectively indicated that the fat-mimetic effect of inulin was highly matrix-dependent, with moderate amounts generally providing the best balance between structural stability, moisture retention and sensory quality, whereas excessive enrichment compromises texture. To preserve product structure and minimize technological losses, the stage at which inulin was added during processing was crucial. The technological stage of incorporation of inulin markedly influenced fiber retention in cheese. In pasta filata (fiordilatte) cheese, the addition of chicory (DP ≥ 23) or cardoon (DP ≥ 50) inulin during the stretching phase minimized fiber loss in-to the whey [52]. In contrast, dissolving inulin directly in milk before coagulation in traditional Giuncata cheese resulted in approximately 20% fiber loss through the whey, consequently reducing cheese yield [13]. A different technological approach has been pro-posed for synbiotic ricotta-type cheese, where 3% long-chain inulin (DP 23–25) and 150 ppm CaCl_2_ were incorporated only after curd separation during homogenization. This approach improved probiotic survival, increased the whiteness of product, and enhanced texture and overall acceptability [45].

In synbiotic dairy products, inulin contributes to the viability and metabolic activity of probiotics during storage. Studies involving strains such as *Lactobacillus acidophilus* and *Bifidobacterium bifidum* have shown that inulin supports probiotic survival, helping to maintain viable cell counts above the recommended minimum level through storage [28,45,46]. The mixture of inulin and calcium chloride further improves the survival of probiotics by stabilizing the protein matrix in cheeses, such as ricotta [45].

#### 4.1.3. Application in Frozen Dairy Products Such as Ice Cream

In frozen (including ice creams, and yogurt–ice cream) and glazed dairy desserts, inulin contributed to foam stability, improved melting characteristics and increased resistance to freeze–thaw cycles, while maintaining a creamy consistency [14]. In those types of products, inulin also primarily functions as a texture stabilizer, fat replacer, and bulking agent. Across different formulations, its addition consistently increased overrun and viscosity while reducing melting rate, thereby improving the physical stability of the products [53,54,55]. However, these technological benefits were dependent on concentration. In low-to-moderate amounts (2–5%), inulin effectively immobilized water within the colloidal gel network, resulting in improved melting resistance and a softer texture. For example, 5% inulin slightly reduced hardness in reduced-fat dairy ice cream [56], whereas 3% inulin improved viscosity, overrun and melting resistance in synbiotic ice cream [54].

**Table 1:** Prebiotic and textural effects of chicory inulin in yogurt.

Product	Inulin Type/Form	Inulin Amount (%)	Technological/Functional Role	Key Processing Conditions	Positive Effects	Limitations	Best-Performing Formulation *	Source
YOGURT								
synbiotic yogurt	inulin powder	1.0–2.0% (+0.5% *S. boulardii*)	prebiotic ingredient; texture and sensory enhancer	fermentation: 43 °C (pH 4.5); storage: 4 °C; 4 weeks	maintained *S. boulardii* viability (>6 log CFU/g); ↑ texture properties; ↑ volatile aroma compounds; ↓ syneresis; denser and more homogeneous microstructure	at 2.0% inulin: ↓ taste, odor and overall acceptability	1.0% inulin (+0.5% *S. boulardii*)	[46]
low-fat synbiotic yogurt	inulin powder extracted from chicory	0.5–2.0%	prebiotic, fat replacer (texture modifier)	inulin extraction: 80, 1 h (1:10 root powder: water); fermentation: 42 °C; storage: 14 days	↑ probiotic viability of *L.* *acidophilus, B. bifidum*, *S. thermophilus* and *L. bulgaricus*; ↑ WHC; ↑ sensory properties; ↓ syneresis; textural properties comparable to full-fat yogurt at 1% inulin	at 2.0% inulin: ↑ pore size; ↓ network firmness	1.0% inulin	[48]
natural yogurt (3.1% fat)	granulated inulin powder	0.0–15.0%	prebiotic functional additive, texture and physical stability enhancer	mixing yogurt and inulin: 20 °C/10 min at 400 rpm; storage: 6 °C/24 h	↑ pH (slight increase at high inulin, 15%); ↑ firmness; ↑ stickiness; ↑ physical stability; ↑ viscosity; ↓ syneresis	high-dose dependent effects (12–15%)	not reported	[10]
probiotic buffalo yogurt	inulin (not specified)	0.2–0.6%	prebiotic and functional ingredient	incubation: 40 °C (pH 4.6); storage: 4 °C/16 days	↑ *B. bifidum* viability (up to 8.83 log CFU/g at 0.6% inulin); ↑ shelf-life; ↓ spoilage microorganisms; ↑ titratable acidity	no significant adverse effects reported	0.6% inulin (optimal; highest *B.* *bifidum* viability and shelf-life)	[57]
probiotic yogurt	commercial inulin and FOS	2.0% inulin + 2.0% FOS	prebiotic and functional ingredient	incubation: 42 °C (pH 4.6); storage: 4 °C; 39 days	↑ shelf-life (39 days); ↑ probiotic survival; ↑ sensory properties; ↑ color perception; ↓ yeast and mold growth	prolonged coagulation time; ↑ titratable acidity during storage (observed in all treatments)	1.0% yogurt starter (1:1) + 1.0% *L. acidophilus* bacteriocin + 2.0% inulin + 2.0% FOS	[16]
stirred bio- yogurt	chicory/artichoke inulin (pure or +MD) and commercial inulin	2.0%	prebiotic and functional ingredient	incubation: 42 °C; storage: 4 °C; 21 days; inulin + MD (ratio 1:2)	↓ syneresis; ↑ viscosity; ↑ sensory properties; ↑ fermentation time (faster acidification); ↑ probiotic survival (>10^6^ CFU/g); denser gel structure	post-acidification during storage (↑ titratable acidity, ↓ pH) and progressive increase in syneresis were observed in all treatments; ↓ L* value	2.0% inulin (chicory/ artichoke): pure for ↓ syneresis; +MD for ↑ viscosity	[28]
yogurt	commercial inulin powder (contains FOS)	1.5%	prebiotic and functional ingredient	storage 1: 0 °C (0–21 days); storage 2: 4 °C (0–7 days)	↑ pH and ↓ titratable acidity compared to control; ↑ LAB growth; potential extended shelf-life	limited sensory panel; single dose	not reported (single inulin amount tested)	[58]

*B. bifidum*—*Bifidobacterium bifidum*; CFU—colony forming units; FOS—fructooligosaccharides; LAB—lactic acid bacteria; *L. acidophilus*—*Lactobacillus acidophilus*; *L. bulgaricus*—*Lactobacillus delbrueckii* subsp. *bulgaricus*; L*—lightness (CIELAB color parameter); MD—maltodextrin; rpm—revolutions per minute; *S. boulardii*—*Saccharomyces boulardii*; *S. thermophilus*—*Streptococcus thermophilus*; WHC—water-holding capacity. * best-performing formulation among the tested treatments. ↑—increase, ↓—decrease.

**Table 2:** Inulin as a fat mimetic and structural stabilizer in cheese.

Product	Inulin Type/Form	Inulin Amount (%)	Technological/Functional Role	Key Processing Conditions	Positive Effects	Limitations	Best-Performing Formulation *	Source
CHEESE								
low-fat synbiotic UF-soft cheese	commercial inulin	1.0–7.0%	fat replacer	heat treatment 75 °C; inoculation with starter culture at 38 °C; coagulation with salt and CaCl_2_ (38 °C/40 min); storage: 7 °C	↑ moisture and ash content; ↑ viability of probiotics; ↑ flavor, body, texture and appearance (7.0% inulin); ↑ creaminess and mouthfeel	↓ protein content and pH; probiotic counts declined during prolonged storage	5.0–7.0%	[49]
imitation mozzarella cheese	commercial inulin	2.4–12.0%	10–50% of vegetable fat replaced	direct steam heating (85 °C) with continuous shearing; emulsification (5 min at 100 rpm)	↓ fat content; ↑ pH; ↑ moisture retention; better stretchability than resistant starch	↑ hardness; ↓ cohesiveness; ↓ springiness; ↓ stretchability at high inulin amounts (vs. full-fat control)	up to 7.2% inulin	[50]
low-fat cottage cheese	commercial inulin	1.0–3.0%	fat replacer	skim milk (0.1% fat); curd inoculated with 2% starter culture; inulin blended into curd; storage at 4 °C for 15 days (analyses on days 1, 7 and 15)	↑ consistency and texture properties; 1.0% inulin produced sensory and physical properties comparable to the 4% fat control	↓ sensory properties and physical properties at 2–3% inulin	1.0% inulin	[51]
fiordilatte cheese (fresh pasta filata)	commercial chicory inulin (DP ≥ 23)/extracted cardoon inulin (DP ≥ 50)	500 g (~5.6% based on curd weight)	fiber enrichment (prebiotic ingredient)	acidification and coagulation 37.5 °C; stretching at 80 °C; inulin added during the stretching phase to reduce fiber loss in the whey; storage: 4 °C for 9 days	↑ texture stability; ↑ color and odor stability; chicory: ↑ overall quality and sensory acceptability; cardoon: ↑ instrumental firmness; ↓ internal color changes	↑ initial growth of spoilage bacteria (*Pseudomonas* spp. for both; Enterobacteriaceae for chicory during the first week)	chicory: slightly better sensory properties; cardoon: stronger texture	[52]
Giuncata cheese (traditional Italian fresh cheese)	commercial inulin	10 g/500 mL (2.0%) and 15 g/500 mL (3.0%)	prebiotic enrichment	inulin powder dissolved in milk before coagulation; curdling: 37 °C/30–40 min	qualified for the EU “source of dietary fiber” claim (>3 g/100 g); ↑ whiteness (L*); minimal effects on pH, color and texture	slight ↓ firmness; fiber loss (~20.0% in whey); ↓ cheese yield at 15 g/500 mL	15 g/500 mL (3.0%)	[13]
synbiotic ricotta-type cheese (from cow’s whey)	long-chain inulin powder (DP 23–25)	1.0–3.0%	synbiotic prebiotic enrichment and probiotic carrier	whey heated to 90 °C and acidified with citric acid; curd homogenized (500 rpm/5 min) with 1–3% inulin and probiotic (10^7^ CFU/g); stored at 4 °C for 5 days	↑ probiotic survival; ↑ texture score; ↑ whiteness (L*); ↑ overall acceptability	↑ titratable acidity and ↓ pH; ↓ protein content	3.0% inulin + 150 ppm CaCl_2_	[45]

CFU—colony-forming units; DP—degree of polymerization; L*—lightness (CIELAB color parameter); ppm—parts per million; rpm—revolutions per minute; spp.—species; UF—ultrafiltration. * best-performing formulation among the tested treatments. ↑—increase, ↓—decrease.

**Table 3:** Cryoprotective and rheological role of inulin in ice cream systems.

Product	Inulin Type/Form	Inulin Amount (%)	Technological/Functional Role	Key Processing Conditions	Positive Effects	Limitations	Best-Performing Formulation *	Source
ICE CREAM								
low-fat yogurt–ice cream	commercial inulin	5.0–9.0%	fat replacer and texture stabilizer	inulin mixed at 45 °C; homogenized (140/35 bar); aged overnight at 5 °C; blended 1:1 with yogurt; hardened at −40 °C (30 min); stored at −18 °C	↑ viscosity; ↑ mouthfeel and sensory properties; 5.0% inulin: slight ↓ hardness; ↓ meltdown rate (gel network immobilize water)	7.0% and 9.0%: ↑ hardness (tougher/denser)	5.0% inulin	[56]
low-fat/sugar synbiotic ice cream (with *B. animalis* subsp. *lactis* and lactulose)	commercial inulin (DP ≥ 23)	5.0%	fat replacer and synbiotic carrier	ingredients mixed at 40–45 °C; aged overnight at 4 °C; hardened at −30 °C (2 h); stored at −18 °C	↑ viscosity and overrun; masks probiotic off-flavor (↑ sensory scores comparable to control); ↑ probiotic survival	↑ hardness when inulin was used alone	technological optimum: 5.0% inulin + *B. animalis* subsp. *lactis* (↑ probiotic count); sensory optimum: 5.0% inulin + 5.0% lactulose (+*B. animalis* subsp. *lactis*) (taste comparable to control)	[53]
yogurt–ice cream (frozen dairy dessert with buttermilk)	dried chicory root extract	2.0–6.0%	low-calorie bulking agent, sweetener and prebiotic supplement	mix pasteurized (80 °C/20 s); 1/3 portion fermented (45 °C/4 h/pH 4.7), blended with rest; homogenized (1000 rpm/45 °C); frozen and stored at −28 °C	↑ color, flavor, texture and sweetness; ↑ overrun; ↓ melting rate; ↑ overall acceptability up to 4.0%	↑ hardness; ↓ overall acceptability if extract exceeds 4.0%	4.0% dried chicory root extract + 0.25% stabilizer + 0.25% emulsifier + 26.43% buttermilk	[59]
synbiotic ice cream	commercial inulin	0.0–3.0%	prebiotic sugar, overrun and viscosity enhancer, and probiotic cell stabilizer	milk heated (50 °C); dry ingredients added; aged at 5 °C/24 h; aerated; stored at −18 °C	↑ overrun, viscosity and thermodynamic stability; ↑ probiotic viability (*L. brevis*); ↑ antioxidant activity; ↓ melting rate and fat destabilization; ↑ color-appearance, flavor, texture and overall acceptance	↑ hardness; ↓ moisture content; risk of curded texture (if total solids are too high) or grainy/icy texture (if total solids are too low)	3.0% inulin + 20.0% sucrose + *L. brevis* PML1	[54]
vegan pea protein ice cream	commercial inulin	2.0–8.0%	fat replacer and prebiotic source	homogenized (3000 rpm/5 min); pasteurized (85 °C/10 min); cooled to 20 °C; aged (4 °C/2 h); frozen (−12 °C/7 min); hardened (−18 °C/14 h)	↑ melting resistance; ↑ overrun (at 2% and 4% inulin); ↓ hardness (improved texture)	↓ sensory scores at high amount of inulin (>4%); ↓ overrun above 4%; ↑ adhesiveness	not reported; 2.0–4.0% inulin showed the best balance between overrun and sensory properties	[60]
fiber-enriched ice cream	commercial inulin	0.7–1.4%	overrun enhancer, texturizer and prebiotic fiber source	soy cream whipped; blended with bacterial cellulose, pulp, and hot water (3.75 HP); dry mix (inulin) added; aged (5 °C/24 h); churned (250 rpm/20 min); stored at −18 °C	↑ sensory score and mouthfeel; ↑ viscosity; ↓ dripping; ↑ overrun (↑ melting resistance); ↑ dietary fiber content	↑ melting rate (17% BC with increasing inulin); ↓ texture and overall acceptability (30% BC)	1.4% inulin + 17% bacterial cellulose	[55]

*B. animalis* subsp. *lactis*—*Bifidobacterium animalis* subsp. *lactis*; BC—bacterial cellulose; DP—degree of polymerization: HP—high pressure; *L. brevis*—*Lactobacillus brevis*; rpm—revoultions per minute. * best-performing formulation among the tested treatments. ↑—increase, ↓—decrease.

Similarly, in low-fat synbiotic ice cream, 5% inulin improved viscosity, overrun and probiotic survival, although it also increased hardness when used without lactulose [53]. Research on yogurt–ice cream and conventional ice cream formulations further confirmed the importance of dose optimization. In buttermilk yogurt–ice cream, the addition of dried chicory root extract up to 4% enhanced sweetness and overall acceptability, whereas higher amounts increased hardness and reduced sensory scores [59]. A similar trend was observed in vegan pea protein ice cream, where 2–4% inulin improved overrun and melting resistance, while amounts above 4% reduced air incorporation and increased adhesiveness [60]. Likewise, in reduced-fat dairy ice cream, increasing the inulin amount to 7–9% resulted in an excessively firm and dense texture [56].

#### 4.1.4. Application in Synbiotic Dairy Products

Although chicory inulin is primarily used for technological purposes to improve product texture, it also serves as an effective prebiotic substrate and carrier for probiotic microorganisms. Numerous studies have demonstrated that its addition significantly increased the survival rate of probiotic cultures and yeasts, such as *L. acidophilus*, *B. bifidum*, *B. animalis* subsp. *lactis*, *L. brevis*, and *S. boulardii*, during long-term storage in a cold or frozen conditions [28,46,53,54,57]. In most formulations, probiotic counts remained above the generally accepted minimum of viable count which is approximately 10^6^ CFU/g throughout storage, supporting the development of functional synbiotic dairy products. For example, Kamel et al. [57] have found that the addition of 0.6% inulin to buffalo milk yogurt ensured high stability of the *B. bifidum* strain (up to 8.83 log CFU/g), which simultaneously resulted in an inhibitory effect on spoilage microorganisms and an increase in product shelf life. Similar findings were reported by Uddin et al. [58], who observed enhanced lactic acid bacteria growth together with a potential extension of yogurt shelf-life following supplementation with 1.5% inulin. The addition of 5% long-chain inulin (DP > 23) significantly increased the survival of *B. animalis* subsp. *lactis* while masking probiotic off-flavors in synbiotic ice cream [53]. Similarly, supplementation with 3% inulin improved the viability of *L. brevis* together with texture and physical stability in synbiotic ice cream [54]. Despite these benefits, increased metabolic activity of probiotic cultures may accelerate post-acidification during storage, leading to progressive pH reduction and increased titratable acidity. Consequently, sensory shelf-life may be shortened because of excessive acidity [16,28]. Additionally, formulations containing high amounts of prebiotic ingredients (2% inulin + 2% FOS) prolonged coagulation time [16], whereas the combination of inulin with maltodextrin reduced L* values during storage of stirred bio-yogurt [28]. In addition, enrichment with sufficient amounts of inulin may enable formulation of products to meet the EU requirements for the nutrition claim “source of dietary fiber” (≥3 g/100 g), as defined in Regulation (EC) No. 1924/2006 [61], in addition to the study on Giuncata cheese. [13].

Besides purified inulin, the application of roasted chicory extracts and whole-root derivatives as multifunctional ingredients was explored. Unlike purified inulin, these materials also provide phenolic antioxidants and characteristic flavor compounds, thereby increasing both the nutritional value and sensory complexity of dairy products. This approach has shown particular potential in ice cream and frozen desserts, where roasted chicory aromas complement the dairy matrix and contribute to improved sensory acceptance [59].

### 4.2. Meat Products

Ingredients derived from chicory, especially inulin, are increasingly used in meat processing technology to improve the nutritional profile and technological properties of this types of products. Due to growing consumer demand for healthier, lower-fat meat products, inulin has been recognized as an excellent functional ingredient that serves as a source of fiber, fat replacer, prebiotic, and texture regulator in meat [14,44,62]. The technological functionality of inulin was primarily manifested through its excellent ability to bind water, stabilize emulsions and form hydrated particle gels whose size was comparable to fat droplets [14,24,63]. This mechanism allowed optimization of rheological properties by increasing creaminess and improving organoleptic properties within low-fat formulations [24]. In addition, incorporation of inulin leads to better moisture retention, higher yield after heat treatment, increased emulsion stability and greater structural strength in a wide range of products such as sausages and burgers [44,64,65,66]. The physical form of inulin significantly affected the texture and structural characteristics of the final product [14,19,24]. While the addition of inulin in the form of powder at higher amounts generally increased hardness, the use of hydrated inulin gels results in a softer texture and more pronounced fat-mimetic properties, which was technologically important, especially in emulsified poultry meat products [24,67]. However, excessive inulin supplementation can impair cohesiveness, elasticity and cutting force in the systems with high moisture content, additionally causing slight changes in pH values and color parameters [24,65].

A review of various studies summarized in Table 4 confirms that inulin, primarily isolated from chicory, is an effective functional ingredient for reformulating various meat products. Its application is most often aimed at reducing the fat content while increasing dietary fiber content and improving the nutritional profile of different meat products. However, the results indicate that the technological and sensory effects of inulin depended on the type of meat products, its physical form, the amount used and the method of incorporation during the technological process.

#### 4.2.1. Application in Various Meat Products

Chicory fibers have been shown to successfully replace part of the lard in recipes for reduced-fat sausages while preserving product cohesion and acceptable color characteristics [62], although in turkey burger formulations, higher amounts of inulin incorporation may result in a darker color of the final product and reduced water-holding capacity [67].

In addition to conventional fat replacement, studies examined the use of inulin in multifunctional reformulation aiming at a simultaneous reduction in fat and sodium chloride content in processed meat products. Inulin has been investigated as a structural component of emulsion gels, which were designed to immobilize vegetable oils within three-dimensional gel networks. These systems enabled the replacement of animal fat with unsaturated oils and improved the nutritional profile of meat products, although their technological performance depended on the type of oil and gel formulation [42]. Product obtained in this way was characterized by a reduced content of saturated fatty acids and cholesterol with improved overall lipid profile. However, the stability of these systems depends mostly on the choice of oil, but also on the ratio between oil and inulin. Among the tested formulations, walnut oil mixed at an oil:water:inulin ratio of 1:2:1 showed the most favorable overall technological performance [42]. In burgers and meatballs, inulin was mainly used as functional supplement or as a partial fat replacer. In most studies, a decrease in fat content and energy value of the final products was observed, together with an increase in moisture or ash content and optimization of key technological parameters, such as water-holding capacity and cooking yield. For example, when used as a functional ingredient rather than a fat replacer, 3% inulin reduced the energy value of chicken burgers by approximately 7% without significantly affecting thermal processing yield [68]. Among burgers, 1% inulin was identified as the optimal formulation under the tested conditions for chicken burgers [68], whereas both 2% and 5% inulin produced acceptable-quality turkey burgers, although the 2% formulation showed more favorable technological properties [67]. In processed meat products, the optimal amount varied considerably, with 6% inulin identified as the most suitable amount for bologna-type sausages [69] and 2.92% inulin enabling 15% fat replacement with the highest overall sensory acceptability in beef and pork frankfurters [65]. At the same time, higher inulin amounts can also be successfully applied depending on the formulation and the desired degree of fat replacement. For example, Kamil et al. [70] demonstrated that beef burgers formulated with 10% inulin, 15% inulin, or 15% inulin combined with 5% residual fat were all considered successful fat-replacement formulations while maintaining acceptable overall sensory and textural quality, despite some differences in individual sensory attributes. Similarly, incorporation of 10% inulin gel as a fat replacer enabled 25% fat replacement in pork meatballs while improving juiciness and overall acceptability [71]. Furthermore, this replacement resulted in higher consumer acceptance than 50% fat replacement in pork meatballs [71]. Similarly, Keenan et al. [64] identified a formulation containing 4.2% fat with 7.3% short-chain and 7.3% long-chain inulin as the optimal balance between technological and sensory quality. Although a completely fat-free formulation containing 18.7% inulin was also technologically feasible, it resulted in greater textural modification than the optimized formulation. In contrast, the use of inulin in powder form at higher amounts was more frequently associated with increased hardness in turkey burgers [67]. A similar trend was observed in emulsified meat products, such as sausages and frankfurters, where inulin was primarily used as a replacement for pork backfat. In such products, its application mainly results in reduced fat content and caloric value, increased moisture content and process yield, and improved water-holding capacity. In chicken frankfurters, 3.67% of inulin was used to replace up to 50% pork fat while maintaining satisfactory technological quality and sensory properties despite substantial fat reduction [72]. By comparing different product groups, it can be seen that coarsely chopped products, such as burgers and meatballs, generally require lower amounts of inulin to preserve desirable properties, while emulsified products allow a higher degree of fat replacement without significant impairment of technological quality. These results suggested that the potential for fat replacement and the acceptable amount of inulin supplementation depended largely on the structure of the product and the complexity of the formulation.

Positive effects have also been reported for emulsified products. In low-fat sausages, replacing fat with short- and long-chain inulin resulted in better emulsion stability and lower cooking losses, while in beef and pork frankfurters, inulin increased process yields while maintaining acceptable technological quality [64,65]. Furthermore, good microbiological stability was observed in bologna sausages during 23 days of storage in a modified atmosphere, accompanied by a simultaneous decrease in the energy value of the product [69].

Similarly, the application of inulin-based emulsion gels enabled the complete replacement of animal fat while preserving acceptable technological properties, although it increased cooking losses and reduced emulsion stability [42]. However, the results were not completely uniform among different formulations. Although inulin generally improved water-holding capacity, processing yield and emulsion stability, these technological benefits were highly dependent on the formulation and were not always accompanied by reduced cooking losses. Its influence on texture was less consistent than its effects on water-holding capacity and process yield, as some formulations showed reduced hardness and improved tenderness [62,65], whereas others became firmer depending on the inulin amount, product matrix and formulation [67,70].

**Table 4:** Application of inulin in meat products: formulation, processing and functional effects.

Product	Inulin Type/Form	Inulin Amount (%)	Technological/Functional Role	Key Processing Conditions	Positive Effects	Limitations	Best-Performing Formulation *	Source
BURGERS								
chicken burgers	commercial inulin (added in rehydrated form—1 part of inulin powder:3 parts of water)	1.0–3.0%	prebiotic ingredient and dietary fiber source	meat ground and mixed; frozen at −28 °C (30 min); grilled at 200 °C to core temperature of 72 °C (7 min); stored for 24 h at 4 °C	↑ sensory quality maintained at 1%; ↓ energy value up to 7% (in sample with 3% inulin); ↑ moisture and ash content	↓ juiciness; ↓ sensory scores for hardness at >1% inulin; slight ↓ protein content	1.0% inulin	[68]
beef burger	chicory root powder	- (defined by fat replacement)	fat replacer (25–75% animal fat replacement)	chicory root: dried at 40 °C for 2 days and milled into powder; burgers: grilled at 220 °C for 8 min	↑ cooking yield; ↓ cooking loss and shrinkage; ↑ crude fiber and ash; ↓ total fat content	lower sensory scores than control, although all formulations remained acceptable (scores > 6.0)	25.0–50.0% fat replacement	[66]
turkey burgers	chicory root inulin powder	2.0 and 5.0%	functional additive/fat replacer (dietary fiber enrichment)	baked at 180 °C to a core temperature of 75 °C; cooled to room temperature	↑ odor and flavor desirability; ↑ structure and cohesiveness	↓ WHC; ↑ hardness; ↓ L* (darkening of cross-section)	not specified; both 2% and 5% inulin produced acceptable- quality products	[67]
beef burgers	inulin extracted from chicory roots	10.0%, 15.0% and 15.0% + 5.0% fat	fat replacer (0% and 5% residual fat)	electric grilling (7–8 min) to a core temperature of 74 °C; frozen storage at −20 °C for 90 days	↑ WHC; ↑ L*; ↓ lipid oxidation during storage	↑ cooking loss; ↑ cooked hardness (with some increases in cooked gumminess/chewiness); ↓ fresh sensory scores (color, flavor, tenderness)	not specified; 10%, 15% inulin and 15% inulin + 5% fat were all considered successful fat- replacement formulations	[70]
MEATBALLS								
pork meatballs	commercial inulin (added as rehydrated powder (1:4 powder-to-water ratio)	10.0% and 20.0% inulin gel	25% and 50% fat replacement	steam-cooked at 100 °C for 15 min; vacuum-packed and stored for 14 days at 4 °C; reheated to 50 °C before evaluation	↓ fat content and energy value; ↑ overall desirability and juiciness during cold storage;	slight ↓ protein content; ↑ water content at 50% fat replacement	10.0% inulin gel (25% fat replacement; higher scores for juiciness, aroma and taste than control)	[71]
pork and chicken meatballs	high-purity inulin powder dissolved in water (1:2 fiber-to- water ratio)	1.8% (+ 1.8% FOS) and 3.5%	fat replacer	boiled at 85 °C to a core temperature of 80 °C; drained for 30 s; subjected to thermal shock to core of 35 °C; packed in modified atmosphere (40% CO_2_/60% N_2_)	↑ internal luminosity (L*); ↓ fat content; high consumer acceptance	↑ cooking weight loss; ↑ sweet flavor intensity; ↓ juiciness; ↑ sensory penalty for “very light color”	not specified; 3.5% inulin showed physicochemical and sensory properties closest to full-fat control	[41]
FRANKFURTERS AND SAUSAGES								
bologna-type cooked sausages	long-chain inulin, commercial (added as frozen gel (1:1 water-to- powder ratio)	3.0–12.0%	fat replacer	cooked to a core temperature of 72 °C; cooled to 6 °C; stored for 23 days at +7 °C	↓ energy content; microbial stability (23 days under MAP storage); citrate mitigated adverse textural effects of inulin; ↓ hardness and gumminess (in citrate batches); acceptable sensory scores (up to 6.0% inulin)	↓ fracturability; ↑ hardness and adhesiveness with increasing amounts of inulin; amounts >6% impaired texture; darker color (especially in phosphate batches); ↑ adhesiveness and slicing difficulties at 12.0%	6.0% inulin + citrate	[69]
breakfast sausages (low-fat)	commercial inulin: long-chain (DP > 23) and short-chain (DP < 10)	0.0–18.7% total inulin (short- and long-chain combinations)	fat replacer (up to 100% fat replacement)	stuffed into casings, cooked in a water bath (85 °C) to a core temperature of 73 °C; frozen storage at −20 °C	↓ cooking loss; ↑ emulsion stability and WHC; acceptable reduced-fat formulations	↑ hardness; ↑ chewiness; ↓ springiness; ↓ sensory juiciness; ↓ perceived saltiness; ↓ overall acceptability at high inulin amounts	two optimized formulations predicted: 4.2% fat + 7.3% short-chain inulin + 7.3% long-chain inulin OR 0.0% fat + 8.3% short-chain inulin + 10.4% long-chain inulin	[64]
frankfurters (beef/pork)	commercial inulin (powder, 92% inulin)	1.46–5.84%	7.5–30.0% fat replacement; texture modifier; prebiotic ingredient	cooked in a water bath to a core temperature of 68 °C; cooled in an ice bath (4 °C for 20 min); drained for 10 min; refrigerated at 4 °C	↑ process yield; ↑ moisture and ash content; ↑ WHC in inulin-pectin formulation; ↓ shear force, hardness, gumminess and chewiness; ↑ sensory acceptance at a moderate level	slightly ↓ color parameters; at the highest inulin amount: ↓ smell, flavor and overall acceptability; ↓ WHC	not specified; T2 (2.92% inulin, 15% fat replacement) showed the highest sensory acceptance and overall acceptability	[65]
restructured pork sausages	chicory fiber powder	10% chicory fiber (replacing 10% pork backfat)	fat replacer	non-smoked: heated at 78 °C to core 75 °C; smoked: drying (50 °C, 25 min), smoking (60 °C, 30 min), heating at 78 °C to core 75 °C; cooled below 10 °C	↓ fat content; ↓ hardness; smoking: ↑ quality characteristics of reduced-fat sausages	↓ moisture; ↓ pH; chicory fiber alone reduced overall quality characteristics	not specified	[62]
chicken frankfurters	commercial inulin (added as gel in a 1:2 inulin powder-to-water ratio)	3.67%	fat replacer (50% pork backfat replacement); component of sodium-reduced formulation	drying (50 °C, 10 min); smoking (60 °C, 30 min); heating at 85 °C to core 72 °C; vacuum-packed and stored at 4 °C for 21 days	↑ moisture; ↓ caloric value and fat; ↑ sensory texture score; improved nutritional profile (↓ sodium, ↑ potassium)	↑ processing and cooking loss; ↓ emulsion stability; ↓ taste score compared with control	not specified (single reformulation tested)	[72]
chicken frankfurters	inulin-based emulsion gels (oil:water:inulin ratios 1:2:0.5 and 1:2:1)	-	full fat replacement	emulsion gel prepared by homogenization and 12 h gelation at 4 °C; drying (50 °C, 10 min), smoking (60 °C, 30 min), cooking to core 72 °C; vacuum storage for 30 and 45 days at 4 °C	↓ fat content; ↑ water and carbohydrate content; ↑ L* and b*; walnut oil provided the best technological performance	↑ cooking loss; ↓ emulsion stability; algal oil formulations produce softer and less cohesive texture	1:2:1 (oil:water:inulin) with walnut oil	[42]

DP—degree of polymerization; FOS—fructooligosaccharides; L*—lightness (CIELAB color parameter); MAP—modified atmosphere packaging; WHC—water-holding capacity. * best-performing formulation among the tested treatment. ↑—increase, ↓—decrease.

The use of inulin in combination with other dietary fibers and hydrocolloids was also investigated. Synergistic systems containing inulin and pectin increased process yield and improved water-holding capacity in reduced-fat frankfurter sausages [65]. However, the technological functionality of inulin largely depended on the used amount, degree of polymerization, physical state and process parameters.

As previously mentioned, inulin was used in products with reduced sodium chloride content; thus, in chicken frankfurters formulated with inulin gel and partial replacement of NaCl with KCl, it was observed that as well as a decrease in sodium content and an increase in potassium content, an improvement in the nutritional profile of the product was achieved [72].

#### 4.2.2. Application in Frozen Meat and Fish Products

In addition to meat products, the stabilizing effect of inulin has also been confirmed in frozen protein-rich systems, such as surimi and fish-based products. In these products, inulin contributes to the preservation of gel strength and protein structure frozen storage, by limiting the growth and recrystallization of ice crystals. This mechanism was attributed to hydrogen-bond formation between inulin, water and protein molecules, which increased the ratio of immobilized and bound water and limited the nucleation and growth of ice crystals during frozen storage [63]. However, during refrigerated storage, fish sausages with added inulin may develop a firmer texture and lower elasticity or juiciness compared to full-fat control samples, although microbial stability and product safety remain uncompromised [43]. In most studies, inulin supplementation improved certain technological properties of the product. For example, beef burgers enriched with chicory root powder showed increased cooking yield and reduced shrinkage after heat treatment [66]. Similarly, beef burgers with 10–15% inulin showed increased water-holding capacity, especially after frozen storage, while formulations with inulin also showed lower thiobarbituric acid values after 90 days at −20 °C [70].

### 4.3. Bakery Products

Products in the bakery industry are often targeted for nutritional improvement because they typically contain a high amount of easily digestible carbohydrates, saturated fats and added sugars, the intake of which is associated with obesity, type 2 diabetes and cardiovascular disease [73]. Consequently, studies and industrial development are increasingly directed toward incorporation of functional ingredients that can enhance the nutritional value of a product while preserving technological quality.

In this context, inulin and fructooligosaccharides (FOS) derived from chicory are widely used as functional ingredients in the production of bread, cakes, biscuits and gluten-free products [15,73,74]. Interest in chicory-derived fructans has grown because these compounds can reduce the caloric value of these types of products (acting as sugar and fat replacers) while simultaneously providing a source of soluble dietary fiber, without impairing texture or sensory properties [8,15,19,75]. In the baking industry, inulin is particularly valued for its thermal stability at temperatures possibly even up to 140 °C, which allows its straightforward incorporation into bread-making processes [74].

The studies summarized in Table 5 show that chicory-derived inulin and related fructans function as multifunctional technological agents in bakery and cereal-based products. Their application contributes to enriching the product with dietary fibers and reducing its energy value, but it also significantly modifies dough rheology, starch digestibility, moisture retention and storage stability. The technological efficiency of inulin largely depends on the degree of polymerization (DP), its physical form and its interaction with starch and gluten, which is why its performance varies among different products and technological systems.

#### 4.3.1. Application in Bread

For the successful formation of gel structures, 20–40% of long-chain inulin is sufficient, while native inulin generally requires a proportion above 30% [74]. Compared with short-chain fractions, long-chain inulin provides better dough stability and has a significantly lower adverse effect on final bread volume [15]. Inulin significantly modified the rheological properties of dough through its high water-holding capacity and gel-forming ability, which directly affected viscosity and may improve gas retention and crumb structure formation [15,19,74]. However, since inulin and gluten proteins compete for available water, an excessive amount of inulin may interfere with proper gluten development. Together with the so-called “gluten-diluting effect”, this resulted in reduced gas retention and the formation of a denser crumb structure [19]. In bread production, the controlled addition of inulin from chicory can improve crumb softness and delay bread staling during storage, while its effect on loaf volume depended on the bread formulation and the type of inulin used [8,15,74]. The addition of 2–4% chicory inulin to wheat bread recapture improved organoleptic properties while contributing to a higher daily intake of soluble dietary fiber [8]. High degree of sucrose replacement with chicory root flour reduced the specific volume of cakes, likely because the lower availability of sucrose limits CO_2_ production during fermentation, as sucrose is the main substrate for yeast activity [75]. Interactions of inulin with gluten are responsible for impact of inulin on bread quality. Addition of inulin improved gluten gel viscosity rather than gluten elasticity, resulting in a soft, sticky inulin–gluten gel. Formation of inulin–gluten gels altered chemical interactions that were present in gluten gels. On one hand, the formation of inulin–gluten gels caused a decrease in ionic bonds, hydrophobic interactions, and disulfide bonds compared with gluten gels without inulin. On the other hand, inulin caused increases in random coil content and decreases in β-sheet content in gluten, while also destabilizing S–S bonds. Through effects on S–S bonds, inulin obstructed the aggregation of gliadin and low-molecular-weight glutenin subunits. The intensity of inulin’s impact on gluten gel properties depended on the DP of inulin, with inulin possessing a higher DP causing more pronounced changes [76].

Due to its pronounced hygroscopic properties, inulin competes with proteins and starch for available water, which directly changes the rheological behavior of the dough. Afshinpajouh et al. [77] showed that chicory inulin in pasta dough increased water absorption and swelling capacity, which result in decreased stability, elasticity and resistance of the dough to stretching. In bread and pastry technology, the addition of oligofructose (5–10%) increased the dough softness and made it more difficult to shape, which requires an adjustment of water, but moderate amounts ultimately resulted in a higher volume and porosity of the baked products [31]. In addition, increased CO_2_ production indicates more intensive fermentation activity and shortening of the proofing time of wheat bread with 2% and 4% inulin [8].

#### 4.3.2. Application in Gluten-Free Bakery Products

Inulin showed exceptional value in the production of gluten-free bakery products, where the lack of gluten proteins creates serious technological difficulties related to dough elasticity and gas retention. In such systems, inulin acts as a structural modifier that partially compensates for the absence of gluten by improving dough and bread structure while enriching the product with dietary fiber. Since it is classified as a food ingredient and not as an additive, its incorporation into gluten-free products provided improved nutritional value while maintaining desirable technological properties. By delaying starch gelatinization and promoting prolonged retention of gases, inulin improved crumb structure and increased the specific volume of rice bread [74].

Burešová et al. [74] showed that gluten-free rice breads, unlike wheat breads, can withstand high amounts of inulin (up to 30%) which acts as a hydrocolloid stabilizer in these systems.

#### 4.3.3. Application in Cakes, Biscuits and Cookies

In cakes and biscuits, inulin is primarily used as a partial fat replacer and as a bulking agent. The use of native and long-chain inulin can form fat-mimetic gel structures that improve mouthfeel and contribute to dough stability and bread structure while reducing the caloric value of the final product [15,19]. However, the addition of excessive amounts of inulin may increase Maillard browning reactions and promote the development of off-flavors because of the reducing properties of inulin, particularly in fine bakery products [15]. In cookies, replacing fat with chicory inulin increased hardness and decreased the spread ratio, particularly at higher levels of fat replacement. Despite these changes, moderate fat replacement generally did not impair the acceptability of sensory properties, although excessive replacement may negatively affect product quality [27].

The effect of inulin depends on the formulation and the ingredients used. For example, in cake formulations, replacing up to 10% of sucrose with chicory root flour as a source of inulin reduced specific volume and produced darker cakes, while increasing specific gravity and hardness [75]. In biscuit technology, partial replacement of fat with inulin (up to 40%) significantly reduced energy value (by up to 7.6%) and increased the content of Fe, Zn and Ca, while maintaining low water activity (a_w_ 0.23–0.25) which ensures microbiological stability [27]. However, high degrees of fat replacement led to a compact structure and increased hardness. When replacing sugar in biscuits, Saumya and Haripiya [78] emphasized the importance of choosing the ingredient itself. While purified commercial inulin allowed high sugar replacement (up to 50%) without loss of quality, roasted chicory root powder can only be used in low amounts (up to 5%) due to the appearance of bitterness and undesirable changes in aroma. A similar pattern was observed when enriching biscuits with fiber. Ivanišová et al. [79] showed that adding chicory fiber extract increased biscuit volume, width and height, while the best sensory properties were achieved at more moderate proportions of 1% to 3%.

#### 4.3.4. Application in Various Cereal Products

Unlike most studies where inulin was added directly to the product formulation, Mariscal-Moreno et al. [80] applied a pre-hydrated inulin paste (40% inulin–60% water) in the production of wheat tortillas. Partial fat replacement (33–66%) with the hydrated inulin paste resulted in softer tortillas, whereas complete fat replacement produced a firmer texture. The use of hydrated paste favored the formation of amylose–lipid complexes and was associated with a softer texture and reduced starch retrogradation. These effects may be linked to the ability of inulin to bind free water and modulate interactions between water, starch and lipids, thereby slowing down product aging during storage. Taken together, the results suggest that the addition of inulin or oligofructose may contribute to prolonging the freshness of various bakery products during storage, as demonstrated by Codină and Bilan [31] on wheat rolls.

The functionality of inulin in cereal products and its ability to stabilize the structure were largely determined by the length of the molecular chain. In pasta production, inulin with a high degree of polymerization (high DP) showed better properties than short-chain fractions. Aravind et al. [39] found that high-DP inulin (at a content of 5%) promoted the formation of a stronger protein-fibrous structure that successfully traps starch granules. This reduced starch digestibility and lowered the predicted glycemic index (GI) of spaghetti.

**Table 5:** Applications of chicory-derived inulin in bakery and cereal-based systems.

Product	Inulin Type/Form	Inulin Amount (%)	Technological/Functional Role	Key Processing Conditions	Positive Effects	Limitations	Best-Performing Formulation *	Source
spaghetti (durum)	commercial inulin (DP ~12–13 vs. DP ~6–7, i.e., higher DP vs. lower DP)	-	0–20% semolina replacement with higher-DP inulin; 0–10% with lower-DP	water adjustment based on farinograph; extrusion and drying; cooking to optimal cooking time	higher DP inulin (5%) ↓ starch digestibility (lower GI); formation of protein-fiber matrix (5%)	20% inulin caused high cooking loss and weak matrix; lower DP negatively affected firmness	5% (higher DP-inulin)	[39]
pasta dough	chicory inulin	1.0–5.0%	-	farinograph, extensograph, and alveograph testing	↑ swelling index (G value); ↑ water absorption	↓ dough stability and elasticity; ↓ resistance to extension	2.5%	[77]
whole meal durum wheat spaghetti	cardoon roots (high DP) vs. chicory (low DP)	4%	-	high DP inulin extraction (DP 50); spaghetti extrusion (1.70 mm)	high DP inulin (from cardoon) is more stable during digestion; ↓ GI in specific cultivars	↑ cooking loss is cultivar-dependent; low DP chicory inulin led to higher loss	not reported (only one amount tested; 4% cardoon-derived high-DP inulin outperformed chicory inulin)	[81]
Turkish noodles	commercial inulin compared with apple, carrot and pea fibers	(defined by flour replacement)	4% flour replacement (dietary fiber enrichment); comparison of different dietary fiber sources	dough rested 25 min; sheeted and cut into noodles; cooking quality, texture and sensory evaluation (ELECTRE)	↑ dietary fiber retained after cooking; ↑ L* vs. other fiber sources; ↓ cooking time; ↑ water absorption; ↑ volume increase; ↓ cooking loss vs. control; similar adhesiveness to control; 2nd highest overall sensory acceptance (after control; highest among fiber-enriched samples)	↓ cooked hardness vs. pea fiber; cooking loss remained higher than pea fiber	not reported (only one amount tested: 4% flour replacement) inulin-enriched noodles were the highest-ranked fiber-enriched formulation according to ELECTRE analysis	[82]
frozen cooked wheat noodles	inulin powder	5% (noodles); 2.5–10% (starch model system)	improvement of frozen storage stability; modulation of starch gelatinization and retrogradation	noodles quick-frozen at −40 °C (50 min), stored at −18 °C for 50 days; cooking, texture and sensory evaluation during storage	↑ starch gelatinization temperature; ↓ peak viscosity; ↓ setback value; ↓ leached amylose; ↓ starch retrogradation; ↓ hardness, viscosity and recooking loss during frozen storage; ↑ sensory acceptability after 50 days	-	not reported (only 5% inulin tested in noodles)	[83]
whole meal durum wheat spaghetti	long-chain Jerusalem artichoke inulin vs. low-chain chicory inulin	- (defined by flour replacement)	2 and 4% flour substitution (dietary fiber enrichment); effect of inulin DP on pasta quality and α-amylase inhibition	whole meal spaghetti produced from two landraces; 2 and 4% flour substitution	↑ elasticity and better maintenance of sensory quality with long-chain inulin; all samples remained sensorially acceptable; ↑ α-amylase inhibition and ↓ IC_50_ with increasing inulin level	↑ water absorption; ↓ swelling index; cooking loss depended on cultivar and inulin type, with greater loss for low-DP chicory inulin; ↓ alanine, leucine, methionine and phenylalanine; ↓ redness and yellowness	no single optimum reported; long-chain inulin performed best sensorially, whereas 4% low-chain chicory inulin showed the strongest α-amylase inhibition in one cultivar	[84]
pasta	commercial inulin (DP ≥ 23)	-(defined by mixture design: 0–10%inulin)	optimization of technological and textural properties; dietary fiber enrichment	mixture design (semolina ≥ 90%; barley flour + inulin ≤ 10%); extrusion; drying at 72.6 °C, 90.98% RH for 6 h	acceptable technological and textural quality; ↓ optimal cooking time; ↑ L*; acceptable firmness and color; few ungelatinized starch granules in cooked pasta	↑ cooking loss; ↓ swelling index; ↓ firmness; ↓ yellowness (b) with increasing inulin level	94.8% durum semolina + 3.7% whole barley flour + 1.5% inulin	[85]
cakes	chicory root flour	-	5.0–10.0% sugar replacement	electric beating (3 min); baking at 210–230 °C	feasibility of prebiotic cakes; maintained hardness and fragility scores similar to control	↓ specific volume; ↑ specific gravity; darker/reddish color	not reported (maximum tested: 10%)	[75]
cookies	chicory root extracted inulin	-	10.0–50.0% fat replacement	creaming (3 min); baking at 200 °C for 7–10 min	↓ energy value (up to 10.2%); ↑ Fe, Zn and Ca contents; low water activity (a_w_ 0.23–0.25) indicating good microbiological stability	↑ hardness; ↓ spread ratio; more compact structure	not reported (acceptable up to 40% fat replacement)	[27]
sweet biscuits	chicory fiber extract	1.0–5.0%	flour replacement (wheat flour enrichment)	roots dried (40 °C); ethanol precipitation; baking at 120 °C	↑ crude protein, ash, and fiber; ↑ volume, width, and height (at 5%); best sensory scores at 1–3% chicory fiber	5% fiber slightly: ↓ sensory acceptability compared to 1–3%	1.0–3.0%	[79]
butter and multigrain cookies	roasted chicory powder/commercial inulin	-	roasted chicory powder: 5–15%; inulin: 5–100% (sugar replacement)	creaming (2–3 min); dough refrigeration; baking at 180 °C	↑ total dietary fiber, ↓ carbohydrate content; roasted chicory improved the mineral profile (Fe, Ca, P); ↑ spread ratio	roasted powder causes bitterness at higher amounts; ↓ cookie volume and weight after chicory/inulin incorporation	5% roasted chicory powder/50% inulin	[78]
rolls (wheat)	oligofructose from chicory root	5.0–10.0%	-	mixing (1–2 min slow + 7–8 min fast); fermentation at 30–32 °C; baking at 250–260 °C for 8–10 min	↑ volume and porosity (91%); ↑ shelf-life (3+ days)	dough become more difficult to mold; required 2–3% additional water (10% oligofructose) and fermentation time had to be adjusted	not reported (10% showed the highest porosity and fiber content)	[31]
wheat bread (first-grade flour)	chicory inulin	2% and 4%	-	fermentation at 38 °C; baking at 220–240 °C	↑ CO_2_ production; ↓ proofing time; ↑ specific volume and freshness	↑ production cost	not reported (2% and 4% were both effective)	[8]
gluten-free rice bread	chicory root inulin powder (DP 2–60)	5–40%	-	proofing 60 min (30 °C, 85% RH); baking 40 min at 180 °C	↑ dough softness; ↓ crumb hardness and chewiness; ↑ specific loaf volume and crumb porosity; ↑ sensory acceptance up to 30%	↑ baking loss; ↓ springiness, cohesiveness and resilience at 40%	30% inulin	[74]
wheat tortillas	hydrated inulin paste (40% inulin/60% water)	-	33.0–100.0% fat replacement	hydration (40% inulin:60% water), 30 min rest, mixing at 40 °C	↑ dietary fiber (up to 57%); ↓ fat (up to 93%); softer tortillas and ↑ extensibility at 33–66%; ↓ starch retrogradation	100% replacement produced firmer tortillas and lighter color; hydrated paste preparation required	33% to 66% inulin paste	[80]

a_w_—water activity; DP—degree of polymerization; GI—glycemic index; IC_50_—concentration required to inhibit 50% of enzyme activity; L*—lightness (CIELAB color parameter); RH—relative humidity. * best-performing formulation among the tested treatment. ↑—increase, ↓—decrease.

In contrast, low-DP inulin negatively affected pasta firmness and increased dry matter loss during cooking. Similar conclusions were reached by Garbetta et al. [81] on whole meal spaghetti, confirming that long-chain fructans were more stable during digestion and that their effect on the glycemic response also depended on the properties of the wheat cultivar itself.

A comparison of long-chain inulin from Jerusalem artichoke and short-chain inulin from chicory showed that a higher degree of polymerization favored the preservation of the sensory quality of whole meal spaghetti, while short-chain inulin was associated with greater cooking loss. In addition, an increase in inulin content resulted in more pronounced inhibition of α-amylase, suggesting a potentially more favorable glycemic response [84].

Recent research indicates that the technological effect of inulin is determined not only by its degree of polymerization, but also by its concentration, the composition of the formulation and the distribution of water within the starch–protein matrix. Increasing the inulin content in pasta often leads to higher water absorption and lower swelling index and firmness, while at higher levels of addition there may be increased cooking loss due to weakening of the gluten network [84,85]. On the other hand, the strong ability of inulin to bind water and form hydrogen bonds can limit the recrystallization of starch and slow down its retrogradation, which is an advantage in products intended for frozen storage [83]. Xu et al. [83] showed that inulin supplementation increases the gelatinization temperature of starch, reduces retrogradation, and slows the increase in hardness and viscosity during frozen storage, resulting in less loss upon recooking and better sensory quality of frozen cooked noodles.

Although inulin supplementation can adversely affect certain technological properties at higher levels of substitution, numerous studies show that with the appropriate selection of inulin type and concentration, products of acceptable or even improved quality can be developed. For example, Zarroug et al. [85] optimized the formulation of a functional pasta containing 1.5% inulin (with 3.7% barley flour) and demonstrated technological and textural properties comparable to the control sample, while Göksel Saraç [82] found that inulin-enriched noodles, among all tested products with added dietary fiber, achieved the highest sensory acceptability after the control sample. This confirms that for the successful use of inulin, it is necessary to find a balance between increasing the nutritional value and preserving the technological and sensory quality of the product.

These conclusions are supported by the review paper by Yazici et al. [86], which shows that the addition of inulin in cereal products, especially pasta, consistently increases dietary fiber content and can help reduce starch hydrolysis and glycemic response. At the same time, the authors point out that the effect of inulin on technological properties, such as optimal cooking time, water absorption, swelling index, texture and cooking loss, is not unambiguous, but depends on the degree of polymerization of inulin, its concentration, type of raw material, and production and processing conditions. As one of the main limitations of the available literature, they highlight the lack of additional in vitro, in vivo and clinical studies needed to better understand the mechanisms of action of inulin and optimize its use in cereal products.

One of the most important mechanisms in interactions between starch and inulin is their competition for water. Since inulin is a hydrophilic molecule, it can bind a considerable amount of water, thereby decreasing the water available for starch granule hydration and gelatinization during cooking. This leads to reduced swelling of starch granules and delayed gelatinization, especially at higher inulin contents [22]. Limited availability of water changes dough rheology properties by increasing dough consistency and reducing starch swelling capacity, resulting in firmer cooked pasta [87]. Interactions between starch and inulin can also occur, and they are primarily of noncovalent nature. The most important interactions are the formations of hydrogen bonds between hydroxyl groups of both polysaccharides. These interactions may restrict amylose mobility during gelatinization, reduce amylose leaching into cooking water, and stabilize the cooked pasta matrix [88].

### 4.4. Functional and Low-Calorie Chocolate Candy Products

In the modern confectionery industry, replacing sucrose is a major technological challenge because, in addition to providing sweetness, it affects the structure, density and rheological properties of the final product [18,89,90,91]. Consequently, multicomponent alternatives are increasingly used in the production of functional and low-calorie chocolates. These most often contain a combination of inulin, polydextrose, intense sweeteners and other bulking agents [91,92,93,94]. Although they are less sweet, inulin and polydextrose successfully replace sucrose because they give the final product volume, texture and good mouthfeel [90,91,92,95]. The functional action of these components primarily results from their ability to modify the rheological and viscoelastic properties of the system, which directly affects the stability and processability of chocolate [90,91,95,96]. For example, incorporating inulin into formulations altered the microstructure of chocolate by changing the organization of fat crystals and the interactions between particles and the fat phase. A higher inulin content increased plastic viscosity and modified yield strength due to particle reorganization and surface changes [91,94,95,96]. These rheological effects were highly dependent on processing conditions, particularly conching temperature, conching time, and particle size distribution [89,96,97]. In their study, Konar et al. [96] targeted particle sizes of 20–25 μm and suggested that reducing the mean particle size may decrease undesirable changes in rheological properties while contributing to a smooth, less grainy chocolate texture. In addition to processing conditions, the degree of polymerization (DP) strongly influenced functional properties, rheological behavior, and sensory acceptability. Long-chain inulin contributes to better structural stability of the system, while short-chain fractions ensure better sensory acceptance by consumers [18,97,98]. In production, inulin is often used in combination with maltodextrin or polydextrose in different ratios. Such formulations affect the crystalline structure of chocolate and, depending on the formulation, can produce textural and sensory properties comparable to conventional products containing sucrose [91,92]. However, caution is required because excessive inulin supplementation can cause discoloration, increased hardness and altered water activity. Therefore, it is crucial to harmonize composition and production in order to achieve an optimal balance between technological quality and nutritional functionality of the product [18,96,97].

The studies summarized in Table 6 showed that chicory-derived inulin acts as a multifunctional ingredient in confectionery products. Depending on the formulation, it can replace sucrose, cocoa butter, or cocoa while simultaneously improving the nutritional profile of the product. However, unlike conventional sweeteners, its technological performance depends on formulation composition, processing conditions, and interactions with other ingredients.

A common feature of most formulations has been that inulin was rarely used as the only replacement for sucrose, but is most often combined with other bulking ingredients or sweeteners to achieve the right balance between the rheological, technological and sensory properties of the product [89,90,92,95,99]. These combinations enable a reduction in sucrose or fat content while maintaining acceptable physical and sensory properties of the product [90,92,99,100]. For example, in milk chocolate, the combination of inulin with polydextrose enabled a reduction in energy value while maintaining hardness and overall acceptability of the product [92], while in sucrose-free chocolate, replacing up to 50% of cocoa butter with inulin (up to 10%) resulted in a product with satisfactory texture and good sensory acceptability [100]. Similar results were obtained by Aidoo et al. [95], who showed that the complete replacement of sucrose requires an appropriate ratio of inulin to polydextrose to achieve suitable rheological properties of chocolate. On the other hand, an increase in inulin content can increase viscosity and change the microstructure of the product, and the sensory properties of the product can also deteriorate at higher proportions [90,99].

In addition to replacing sucrose and fat, ingredients derived from chicory can also be used as partial replacements for cocoa. Fadel et al. (2006) [101] showed that the combination of roasted chicory root and carob produces an aromatic profile similar to cocoa, although it differs in the intensity of individual aromas. Similarly, the use of chicory root powder as a partial replacement for cocoa also influenced the physicochemical, rheological and sensory properties of waffle cream, confirming that the choice and ratio of replacer ingredients significantly determined the final quality of the product [102]. Overall, the success of reformulation depended primarily on the interaction of inulin with other ingredients in the formulation and the selection of an appropriate combination of replacer ingredients.

Changes in rheological properties represent one of the most important technological challenges in the application of inulin in confectionery products. The analyzed studies show that the effect of inulin on viscosity, yield strength, hardness and microstructure is not consistent across studies, but depended on the interaction between the used amount, degree of polymerization, formulation composition and production conditions. Consequently, products with the same inulin content can exhibit different rheological properties [89,96]. For example, Konar [89] showed that conching temperature significantly affected the rheological properties of milk chocolate, with the choice of sweetener further modulating these properties. Several studies have shown that inulin supplementation changed the microstructure and distribution of the fat phase within the chocolate mass, which is directly reflected in the rheological behavior of the product. However, the direction of these changes was not always the same. Shourideh et al. [99] and Rodriguez Furlán et al. [90] reported an increase in apparent viscosity and yield strength at higher inulin proportions. Rodriguez Furlán et al. [90] also showed that increasing the inulin content leads to the formation of a denser crystal network and increased thermal stability and resistance to fat bloom. Conversely, Konar et al. [96] found that with certain combinations of inulin amounts and process conditions, viscosity, yield strength and hardness can be reduced. Particle size plays a particularly important role, as it directly affects product smoothness and the stability of the chocolate mixture. Reducing the mean particle size increased the specific surface area and the requirement for free fat needed for complete coating of the particles. When the amount of free fat was insufficient, viscosity increased, microstructure changed and the sensory properties of the product deteriorated [96,100]. Therefore, the successful application of inulin cannot be based only on the selection of the appropriate amount, but requires the optimization of process parameters, especially particle size, conching and tempering.

In addition to its technological role, inulin in confectionery products was also an important functional ingredient due to its prebiotic properties. Although most research emphasized its effect on rheological properties, some authors have investigated the possibility of developing synbiotic products by combining inulin with probiotic microorganisms. This approach enables the development of functional confectionery products that, in addition to satisfactory technological properties, may also provide potential health benefits due to the combination of the prebiotic effect of inulin and the viability of probiotic cultures [98,103].

**Table 6:** Applications of chicory-derived inulin in functional and low-calories chocolate candy products.

Product	Inulin Type/Form	Inulin Amount (%)	Technological/Functional Role	Key Processing Conditions	Positive Effects	Limitations	Best-Performing Formulation *	Source
cocoa substitute	roasted chicory roots + carob pods (1:2)	-	cocoa replacement	milling; roasting at 170 °C for 25–30 min; milling and nitrogen packaging	↑ sensory similarity to cocoa; comparable chocolate aroma; key cocoa volatiles identified; ↑ chocolate aroma after 6-month storage	↓ cocoa-like and sweetish/caramel notes; ↑ earthy/roasted notes	not reported (single ratio tested 1:2)	[101]
chocolate mousse	commercial chicory inulin	50.10 g/kg	prebiotic (synbiotic) ingredient	mixing; heating to 80–85 °C; cooling to 40 °C; inulin addition; whipping until volume doubled; storage at 4 °C	↑ firmness and adhesiveness; stable probiotic viability; favorable texture	altered color perception; taste, aroma and texture differed from control	not reported (single amount tested: 50.10 g/kg)	[103]
milk chocolate	commercial chicory inulin	0–100% of the sugar substitute blend	sucrose replacement (inulin, polydextrose, maltodextrin + 0.04% sucralose)	particle size reduction (20–38 μm); conching for 3.5 h at 65 °C (50 rpm); lecithin addition; conching for 30 min; overnight storage at 50 °C; tempering (20 → 30 °C); cooling at 15 °C	↓ energy content; ↑ overall acceptability with high inulin/polydextrose ratios; hardness comparable to the control with 100% inulin; feasible production of prebiotic low-sugar milk chocolate	↑ moisture with high polydextrose/ maltodextrin; ↓ melting rate, mouth coating and overall acceptability with high maltodextrin; ↓ sensory quality when fat reduction exceeded 5%	inulin: 14–32% or 71–84% of the sugar substitute blend	[92]
dark chocolate	commercial inulin	0.0–51.4% (within sucrose replacer blend)	sucrose replacement (inulin:D-tagatose blends)	ball milling (85 rpm, 50 °C, 3 h); drying at 50 °C for 12 h; tempering (75 rpm); stevia added to adjust sweetness	↓ energy (up to 27.5%); darker color; ↓ yield stress; prebiotic potential	↑ apparent and plastic viscosity; ↓ sensory acceptance at high inulin amounts	25% inulin: 75% D-tagatose or 100% D-tagatose	[99]
milk chocolate	commercial chicory inulin	9.0%	partial sucrose replacement using maltitol or isomalt	mixing at 40 °C; pre-refining (20 μm); dry conching (45 min); conching 270 min at 50, 55 or 60 °C; three-stage tempering (33–35 → 24–25 → 25–26 °C); cooling at 5 °C	↓ water activity; maltitol produced color, hardness and rheological properties closest to sucrose-containing chocolate; acceptable sugar-free prebiotic milk chocolate	rheological properties strongly depended on conching temperature; isomalt showed higher viscosity and may produce pasty mouthfeel	not reported (single amount tested: 9% inulin; maltitol identified as the preferred sweetener)	[89]
sugar-free dark chocolate	commercial chicory inulin	0–100% inulin (within polydextrose)	complete sucrose replacement	mixing ingredients; three-roll refining (28–30 μm); dry conching for10 min at 65 °C; added cocoa butter and lecithin; wet conching for 15 min at 50 °C → tempering; cooling	acceptable rheological and physical quality at the optimum formulation; darker color than control	Casson plastic viscosity depended on the inulin/polydextrose ratio; Casson yield stress depended on formulation; inulin alone produced very high viscosity and should be combined with polydextrose	24.64% inulin: 75.36% polydextrose	[95]
milk chocolate	commercial chicory inulin	60, 90, 120 g/kg	prebiotic dietary fiber; low-calorie bulking agent	three-roll refining (20, 25 or 28 µm); conching for 3.5, 4.0 or 4.5 h at 55 °C; three-stage tempering (33–35 → 24–25 → 25–26 °C)	↓ water activity; target particle size (20–28 µm) successfully achieved at all inulin levels	↑ specific surface area and D_90_; rheological properties strongly depended on refining and conching conditions	not reported	[96]
sucrose-free chocolate	commercial chicory inulin	3.2–10%	partial cocoa butter replacement (up to 50%)	mixing at 40 °C (10 min); refining to 17–23 μm; conching for 14 h at 60 °C; tempering (50→27→30 °C); cooling to 11 °C	↓ hardness; maintained good sensory acceptance; ↑ mouthfeel and sensory acceptance compared with β-glucan; successful fat reduction while preserving chocolate quality	high inulin amounts slightly ↓ texture and flavor acceptance; altered chocolate microstructure due to incomplete coating of solid particles	10% inulin (10 g/100 g chocolate; replacing 50% cocoa butter)	[100]
white compound chocolate	inulin	5.0% and 10.0%	surfactant/ stabilizing agent	extrusion 1 h at 35 °C; conching 7 h at 45 °C (200 rpm); tempering (23–24 °C cooling → 28–29 °C heating); cooling 2 h at 7 °C	5%: ↑ shelf-life; ↑ whiteness; ↓ apparent viscosity (lubricating effect); 10%: ↓ free fat release; ↑ apparent viscosity and yield stress; denser crystal network; ↑ thermal stability; ↓ fat crystal size; ↑ bloom resistance	5%: ↓ viscosity may promote fat migration; 10%: ↑ viscosity and yield stress	not reported: 5% improved shelf-life; 10% for structural stability	[90]
probiotic white chocolate	inulin (low DP <10; high DP >23)	9.0% (9 g/100 g)	bulking agent/prebiotic (partial replacement of sucrose or maltitol)	mixing at 40 °C; pre-refining; dry conching 45 min; wet conching (total 360 min at 60 °C); probiotic addition (9 log CFU/25 g) at 35 °C; tempering 33–35 → 24–25 → 25–26 °C; cooling 20 min at 5 °C	↑ probiotic viability for 90 days (>6 log CFU/25 g); ↑ survival of *L. acidophilus* vs. *L. paracasei*; DP had no significant effect on probiotic viability; ↑ hardness in some sucrose formulations; acceptable water activity (<0.4); effects on quality parameters were generally tolerable	↑ plastic viscosity and yield stress in some formulations; higher-DP inulin ↑ hardness with high DP in sucrose chocolate; quality changes depended on sweetener type	not reported (single amount tested: 9% inulin)	[98]
cream waffle	chicory root powder	0–20% (0, 3.33, 6.67, 10, 13.33, 20% of cocoa powder replacement; alone or combined with carob powder)	cocoa powder replacer	ingredients mixed sequentially using a hand mixer: oil (1–2 min low speed, 2–3 min high speed), sugar added and mixed (1–2 min), followed by milk powder and cocoa/carob/chicory powders (2–3 min; samples stored and evaluated after 1, 30 and 60 days	↓ caffeine, peroxide value; ↑ antioxidant activity; ↑ sensory acceptance and physicochemical properties; modified rheological properties	modified viscosity, color and sensory attributes; ↓ rheological properties with increasing carob powder substitution	two formulations: (1) 20% carob powder showed favorable physicochemical characteristics; (2) 10% cocoa + 10% carob powder achieved the highest sensory acceptance	[102]

CFU—colony forming units; D_90_—particle diameter below which 90% of particle volume is distributed; DP—degree of polymerization; *L. acidophilus*—*Lactobacillus acidophilus*; *L. paracasei*—*Lactobacillus paracasei*; rpm—revolutions per minute. * best-performing formulation among the tested treatments. ↑—increase, ↓—decrease.

The results of available research show that confectionery matrices, especially chocolate, can be a suitable carrier for probiotic cultures due to their low water content and high fat content, which contributed to their stability during storage. In the synbiotic chocolate mousse, the addition of commercial inulin made it possible to maintain high viability of probiotic bacteria while increasing the firmness and adhesiveness of the product, although minor changes in the perception of color, taste and aroma were noted compared to the control sample [103]. Similarly, in probiotic white chocolate, the addition of 9% inulin made it possible to maintain the number of probiotic cells 6 log CFU/25 g for 90 days of storage, with *Lactobacillus acidophilus* showing greater viability than *Lacticaseibacillus paracasei*, while the degree of inulin polymerization did not significantly affect the viability of probiotic cultures [98].

Despite these promising results, the development of synbiotic confectionery products requires careful optimization of the formulation. The synbiotic combination of inulin and probiotic cultures can affect the rheological properties of products, including an increase in plastic viscosity, yield strength and hardness of individual formulations, where the intensity of changes depends on the type of sweetener and the degree of polymerization of inulin [98]. Previous research shows that chocolate represents a promising matrix for the development of synbiotic products, where the key compromise is between preserving the technological quality of the product and maintaining the high sustainability of probiotic cultures.

## 5. Practical Relevance

This review summarized the application of inulin in the formulation of various types of food products, showing its versatile use due to its structure and resulting properties. This summary provides a valuable platform for future food product development as well as for the improvement of existing products. The technological properties of inulin are highly important, but its impact on health is also significant. Greater consumer awareness of the impact of food on health—i.e., eating not just for pleasure and hunger satisfaction, but also to nourish the body—has led to healthier choices. However, consumers still demand sensorially appealing food in addition to health benefits. Both of these factors can be influenced by adding inulin to food products; inulin can be assessed for improving food product quality from both technological and nutritional points of view.

The many health-promoting benefits of inulin were reviewed in detail by Alonso-Allende et al. [104]; however, its prebiotic effect is probably the best known among consumers. Considering the various food products that can be prepared by adding inulin, the question remains how much inulin is present in the final product, and whether it is more likely to be present in the form of FOS and OF (fructooligosaccharides and oligofructose, respectively). Studies have shown that these compounds affect the gastrointestinal tract, and in a systematic review of these results by Mysonhimer and Holscher [105] it was concluded that the recommended dose of inulin is up to 5 g/day to avoid more than mild flatulence. However, this is quite individual, and it was concluded that doses up to 20 g/day can be well tolerated by some individuals, aside from mild-to-moderate bloating and flatulence. Therefore, although a therapeutic dose of up to 15–20 g/day of inulin to improve laxation would likely result in some intolerance symptoms, they would likely be only mild or moderate. For FOS and OF, the recommended intake is 7.8 g/day to avoid intolerance symptoms. However, to achieve a therapeutic effect, a dose of 10–15 g/day to improve mineral absorption would still be well tolerated, with likely only mild flatulence [105]. Beyond its prebiotic effect, a less widely known effect among consumers is its impact on blood glucose levels, which is a key factor for metabolic health. Inulin intake can reduce blood insulin concentrations and improve insulin sensitivity. An inulin intake of 10 g/day for 8 weeks caused a decrease in blood glucose concentrations and improved overall glucose metabolism [104]. This is just one example; however, it has been shown that inulin can contribute to this effect across a wide range of dosages and different intervention periods. Additionally, it has been demonstrated that inulin can reduce total cholesterol and triglyceride concentrations in blood at different doses [104]. Even though the amounts of inulin added to different food products may not have an immediate impact on health, its long-term effects on health could be observed, regardless of whether it is present as inulin or in the form of its degradation products.

## 6. Conclusions, Future Perspective and Limitations

Inulin derived from chicory root and commercial inulin has wide application in the food industry due to its modification of quality properties. Its most extensively studied use is in dairy products, but it is also widely used in meat and bakery products, as well as functional and low-calory chocolate candy products. Its techno-functional behavior in foods is strongly related to the degree of polymerization, and, depending on this, it can be used to modify texture, replace fat and sugar, enhance probiotic viability and improve the nutritional value of foods. Its added amount is another key factor influencing its behavior in foods; in addition, its form and the main processing conditions are also very important. Besides techno-functional behavior, it is necessary to consider its impact on sensory properties, so a balance must be achieved when selecting the amount, form, and processing conditions to obtain the desired quality of food products. Due to its prebiotic properties, inulin is an important ingredient for enriching food products with fiber; however, its final form, i.e., presence in the final product as inulin or lower-molecular degradation products remains questionable.

Generally, the application of inulin in the future has high potential not just from a technological point of view but also due to its health-promoting properties; thus, its importance in the formulation of new food products, especially functional ones, is on the rise. However, it is to be expected that the rise in its application in low-fat, low-sugar, gluten-free products will be significant, especially since it is a plant-based ingredient. Due to its properties as well as interactions with other compounds, it is to be expected that its application in the formulation of edible coatings and films, as well as encapsulating material for various bioactive and flavor compounds, will be significant. Thus, overall, inulin is a valuable polymer with multiple functions which can be used in food production, but these properties could also be used in the pharmaceutical, cosmetic, agricultural, and medical industries.

Some limitations in inulin’s application in various products do exist and should be taken into account when formulating new food products or improving existing ones. One has already been mentioned: presence of inulin in the final product, that is, its form—as intact inulin or lower-molecular-weight degradation products. In addition to its form in the final product, the question is whether the amounts of inulin and lower-molecular-weight degradation products will have a health-promoting impact on the body. It is very likely that an immediate impact on health will not be observed; however, clinical studies have shown that long-term effects on health can be seen with continuous consumption of these products. Another limitation is the need to balance desirable properties and health benefits with the sensory appeal of the final food products. Ultimately, these so-called healthy food options need to have a pleasant flavor, since this is a highly significant product quality attribute that affects consumer acceptance of the product and purchase decisions. Quite often, consumer selection and commitment to innovative, new food products depend heavily on flavor, and this attribute is also a key factor in motivating regular consumers to modify their usual eating patterns.

## Data Availability

No new data were created or analyzed in this study. Data sharing is not applicable to this article.
